# Pregnancy After Bariatric Surgery—Experience from a Tertiary Center

**DOI:** 10.1007/s11695-024-07147-y

**Published:** 2024-03-11

**Authors:** Helena Urbano Ferreira, Madalena von Hafe, Helena Dias, Juliana Gonçalves, Sandra Belo, Joana Queirós

**Affiliations:** 1 Serviço de Endocrinologia, Diabetes e Metabolismo, Centro Hospitalar Universitário de São João, Alameda Prof. Hernâni Monteiro, 4200-319 Porto, Portugal; 2 Instituto de Investigação e Inovação em Saúde (i3s), Faculdade de Medicina da Universidade Do Porto, Porto, Portugal; 3 Serviço de Pediatria do Centro Hospitalar Universitário São João, Porto, Portugal; 4 Serviço de Obstetrícia do Centro Hospitalar Universitário de São João, Porto, Portugal

**Keywords:** Pregnancy, Bariatric surgery, Metabolic surgery, Obesity

## Abstract

**Introduction:**

It is estimated that most people undergoing bariatric surgery are women of reproductive age; nonetheless, its effects on pregnancy outcomes are not yet fully understood.

**Methods:**

Retrospective observational study, conducted in a tertiary center in Portugal, included participants in two groups: (1) pregnant women with a history of bariatric surgery (*n* = 89) and (2) pregnant women with a BMI ≥ 35 kg/m2, without previous bariatric surgery (*n* = 176). Data was collected from the medical files. Multivariate analysis was conducted to adjust for confounders.

**Results:**

Pregnancy after bariatric surgery was associated with lower risk of gestational diabetes (15.7% vs. 30.1%, *p* = 0.002) and cesarean delivery (20.7% vs. 33.5%, *p* = 0.007), and a higher gestational weight gain (10.58 ± 9.95 vs. 7.33 ± 6.00 kg, *p* < 0.001). Participants in the bariatric surgery who experienced a gestational weight gain ≤ 10.0 kg had a higher risk of preterm delivery (16.7% vs. 2.5%, *p* = 0.031). No significant differences were found regarding hypertensive diseases of pregnancy between groups (4.5% vs 11.4%, *p* = 0.147).

Pregnancy after bariatric surgery was associated with lower neonate weight percentile (34.24 ± 21.09 vs. 48.77 ± 27.94, *p* < 0.001), higher risk of fetal growth restriction (5.6% vs. 0.6%, *p* = 0.018), and lower risk of fetal macrosomia (0.0% vs. 7.5%, *p* = 0.005). There were no significant differences in the risk of SGA (12.5% vs. 7.0%, *p* = 0.127) or LGA neonates (2.3% vs. 6.4%, *p* = 0.069).

**Conclusion:**

Pregnancy after bariatric surgery is associated with both risks and benefits, which should be considered by healthcare providers. Pregnancy after bariatric surgery requires individualized care, to ensure adequate gestational weight and avoid micronutrient deficiencies.

**Graphical Abstract:**

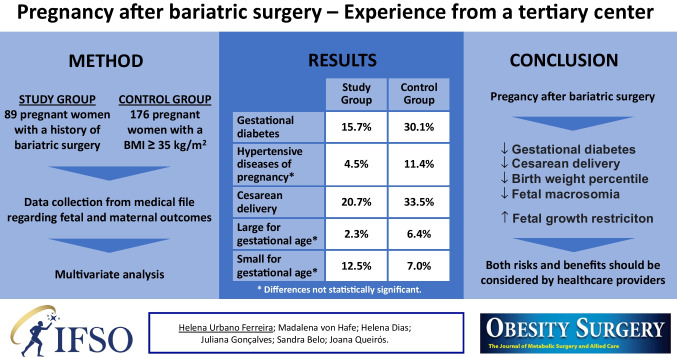

## Introduction

### Background

Maternal obesity is associated with adverse outcomes for both the mother and fetus. Women with obesity before pregnancy are at increased risk of gestational diabetes, preeclampsia, preterm delivery, stillbirth, and congenital anomalies [1, 2]. Additionally, children born to mothers with obesity are more likely to develop obesity, metabolic syndrome, and cardiovascular disease later in life [1, 3, 4].

Bariatric surgery is a safe and effective procedure and is currently the most effective treatment for obesity, resulting in significant and sustained weight loss and remission of comorbidities [5, 6]. It is estimated that about 80% of people undergoing bariatric surgery are women, many of whom are of reproductive age [7], and an increasing number of pregnancies after bariatric surgery have been observed over the last decades [8]. Nonetheless, the effects of bariatric surgery on pregnancy outcomes are not yet fully understood.

Previous studies have shown that, on the one hand, bariatric surgery before pregnancy leads to a reduction of complications associated with maternal obesity, namely, gestational diabetes, and hypertensive disorders of pregnancy [9–11]. On the other hand, it has also been associated with inadequate gestational weight gain, newborns small for gestational age (SGA), fetal growth restriction, and micronutrient deficiencies [9–13].

The optimal timing of pregnancy after bariatric surgery remains a topic of debate. It is usually recommended waiting for 12–24 months after surgery before attempting to conceive [8, 14, 15]. In addition, some studies point to malabsorptive surgeries (gastric bypass) being associated with worse maternal and fetal outcomes [16].

### Aim

The primary aim of this study was to compare maternal and fetal outcomes in two groups: a first group of women who underwent bariatric surgery before pregnancy and a second group of women with obesity class II or III, without prior history of bariatric surgery.

The secondary aim was to compare maternal and fetal outcomes within the group of women who underwent bariatric surgery prior to pregnancy, based on the type of surgery performed, time between surgery and pregnancy, and gestational weight gain.

## Methods

### Study Design

This was a retrospective observational study, evaluating pregnant women followed by a multidisciplinary team, including obstetricians, endocrinologists, and nutritionists specialized in maternal obesity, in a tertiary hospital in Portugal, from September 2019 to September 2022.

### Study Participants

#### Inclusion Criteria

Participants were included in two groups. The first group included women ≥ 18 years old, who underwent either sleeve gastrectomy or gastric bypass before pregnancy. The second group, also referred as the control group, included women with a body mass index (BMI) ≥ 35 kg/m^2^.

#### Exclusion Criteria

Twin pregnancies were excluded as well as participants with loss of follow-up during pregnancy.

### Data Collection

Data was collected retrospectively from the participants’ medical files. Information was collected regarding participants sociodemographic characteristics, past medical history and habits, medication during pregnancy, routine blood analysis, gestational weight gain, type of delivery, newborn anthropometry, and fetal and maternal complications. Clinical definitions and diagnostic criteria considered in this study are described in Table 1.

**Table 1:** Clinical definitions and diagnostic criteria considered in this study

Body mass index (BMI)	Weight (kg)/(height (m))^2^
Maternal age	Maternal age at delivery
Gestational age	Crown-to-rump length measured in the first trimester ultrasound performed between 11 and 13 + 6 weeks of gestation [17]
Gestational weight gain	Difference between reported prepregnancy weight at weight in last prenatal visit just prior to delivery
Gestational diabetes	Criteria used in the control group: • Fasting glucose in the first trimester ≥ 92 and < 126 mg/dl • Two-hour 75g OGTT performed at 24 to 28 weeks of gestation. Gestational diabetes is diagnosed if one value is altered: fasting plasma glucose ≥ 5.1 mmol/L; 1-h plasma glucose ≥ 10 mmol/L; or 2-h plasma glucose ≥ 8.5 mmol/L) [18] Criteria used in the bariatric surgery group: • Fasting glucose in the first trimester ≥ 92 and < 126 mg/dl • Capillary blood glucose monitoring for 1 week between 24 and 28 weeks of gestation. Gestational diabetes is diagnosed if values are above target: fasting and preprandial blood glucose ≥ 5.3 mmol/L; 1-h postprandial blood glucose ≥ 7.8 mmol/L; 2-h postprandial blood glucose ≥ 6.7 mmol/L) [14]
Essential hypertension	Systolic blood pressure ≥ 140 mmHg and/or diastolic blood pressure ≥ 90 mmHg on two occasions (at least 4 h apart), before pregnancy or present before 20 weeks of gestation [19]
Gestational hypertension	Systolic blood pressure ≥ 140 mmHg and/or diastolic blood pressure ≥ 90 mmHg on two occasions (at least 4 h), apart after 20 weeks of gestation, in a woman with a previously normal blood pressure [20]
Preeclampsia	New-onset hypertension, after 20 weeks of gestation, accompanied by end organ lesion (most often new onset proteinuria) [20]
Fetal growth restriction	Abdominal circumference(AC)/estimated fetal weight(EFW) < 3rd percentile OR AC/EFW < 10th percentile and at least one of the following: 1. AC/EFW crossing centiles > 2 quartiles on growth centiles* 2. Cerebroplacental ratio < 5th centile 3. Umbilical artery pulsatility index > 95th centile 4. Uterine artery pulsatility index > 95th centile [21]
Small for gestational age	AC/EFW < 10th percentile without changes in Doppler fluxometry [21]
Large for gestational age	EFW or AC > 90th percentile [22]
Fetal macrosomia	Birth weight > 4000 g [22]
Labor dystocia	Abnormal labor progression during the latent (up 6-cm dilation) or active phases (from 6 cm until full dilation) of the first stage of labor, or during the second stage (from complete cervical dilation until delivery of the fetus) [23]

### Statistical Analysis

The data collected in this study was analyzed using SPSS® Statistics version 27. Descriptive statistics, including mean, standard deviation, median, and interquartile range, were calculated for continuous variables, as appropriate, while frequencies were calculated for categorical variables. Normality of data distribution was examined using skewness and kurtosis. Bivariate analysis was conducted using *t*-tests, Mann–Whitney *U* tests, or correlations for continuous variables, as appropriate, and chi-square and Fisher’s exact tests for categorical variables. Multivariable logistic and linear regression analyses were used to adjust for possible confounders such as BMI before surgery in the bariatric surgery group and BMI before pregnancy in the control group, maternal age, number of previous gestations and abortions, history of active smoking during pregnancy, and essential hypertension. The statistical significance level was set at *p* < 0.05, and all tests were two-tailed. Statistical assumptions for statistical tests were evaluated, and no violations were detected.

## Results

### Participant Characteristics

Eighty-nine gestations of 74 women were included in the bariatric surgery group, 70.8% of whom had undergone gastric bypass, and 176 women were included in the control group (Table 2). Participants in the bariatric surgery group were older (32.9 ± 4.5 vs. 31.3 ± 5.7 years, *p* = 0.017), more likely to be married (76.6% vs. 61.1%, *p* = 0.012), and had a lower BMI before pregnancy (30.0 [22.4–37.5] vs. 38.3 [33.0–43.6] kg/m^2^, *p* < 0.001). No statistical differences were found between groups regarding educational level, number of previous gestations and abortions, history of essential hypertension, and active smoking during pregnancy.

**Table 2:** Participants’ characteristics

	Bariatric surgery group (*n* = 89)	Control group (*n* = 176)	*p* value
Maternal age—years, mean ± SD	32.9 ± 4.5	31.3 ± 5.7	0.017
Marital status—*n* (%)			
Single	18 (20.9%)	63 (36.0%)	0.013
Married^1^	66 (76.6%)	107 (61.1%)	0.012
Divorced	2 (2.3%)	5 (2.9%)	1.000
Educational level—*n* (%)			
Primary education (1–9 years)	25 (28.7%)	57 (32.4%)	0.548
Secondary education (10–12 years)	47 (54.0%)	89 (50.6%)	0.598
Superior education (≥ 13 years)	15 (17.2%)	30 (17.0%)	0.968
Type of bariatric surgery—*n* (%)			
Gastric bypass	63 (70.8%)	-	-
Sleeve gastrectomy	26 (29.2%)	-	-
Time from bariatric surgery to pregnancy—months, mean ± SD	44.0 ± 31.5	-	-
BMI before bariatric surgery—kg/m^2^, mean ± SD	43.9 ± 5.2	-	-
BMI before pregnancy—kg/m^2^, median (IQR)	30.0 (7.5)	38.3 (5.3)	< 0.001
Previous gestations—*n* (%)			
1	27 (30.3%)	60 (34.1%)	0.539
2	33 (37.1%)	64 (36.4%)	0.909
≥ 3	29 (32.6%)	52 (29.5%)	0.612
Previous abortions—*n* (%)			
0	72 (80.9%)	139 (79.0%)	0.714
1	14 (15.7%)	29 (16.5%)	0.876
≥ 3	3 (3.4%)	8 (4.5%)	0.756
Smoking during pregnancy—*n* (%)	9 (10.1%)	20 (11.4%)	0.758
Essential hypertension—*n* (%)	5 (21.7%)	18 (10.2%)	0.208

^1^Including common-law marriage. *SD* = standard deviation. *IQR* = interquartile range

### Maternal and Fetal Outcomes

Participants in the bariatric surgery group had a higher mean gestational weight gain (10.58 ± 9.95 vs. 7.33 ± 6.00 kg, *p* < 0.001), higher frequency of eutocic delivery (69.0% vs. 44.5%, *p* > 0.001), and lower frequency of cesarean delivery (20.7% vs. 33.5%, *p* = 0.007; Table 3). Among participants who had a cesarean delivery, there was a tendency to a lower frequency of labor dystocia in the bariatric surgery group (5.6% vs. 29.8%, *p* = 0.055; Table 4). A lower frequency of induced labor was observed in the bariatric surgery group (33.3% vs. 48.0%, *p* = 0.025), but statistical significance was lost after adjusting for covariates (*p* = 0.065). There was no difference in the duration of hospitalization between groups (3 vs. 3 days, *p* = 0.083).

**Table 3:** Comparison of maternal and fetal outcomes among the bariatric surgery group and the control group

	Bariatric surgery group (*n *= 89)		Control group (*n* = 176)		Bivariate analysis			Multivariate analysis^1^		
					Mean/median difference		*p* value	Beta coefficient		Adjusted *p* value
Gestational weight gain—kg, mean ± SD	10.58 ± 9.95		7.33 ± 6.00		3.25		< 0.001	3.686^2^		< 0.001^2^
Gestational age at birth—weeks, median (IQR)	39.07 (1.86)		39.57 (1.21)		-0.65		0.002	− 3.740		0.007
Newborn weight—percentile, mean ± SD	34.24 ± 21.09		48.77 ± 27.94		-14.53		< 0.001	− 16.600		< 0.001
Newborn length—percentile, mean ± SD	38.09 ± 21.21		51.47 ± 23.33		-13.38		< 0.001	− 17.123		< 0.001
Newborn head circumference—percentile, mean ± SD	43.81 ± 26.60		55.29 ± 29.57		-11.48		0.002	− 14.683		< 0.001
Hospitalization duration—days, median (IQR)	3 (1.3)		3 (1.0)		0		0.083	− 0.486		0.077
	Bariatric surgery group (*n* = 89)		Control group (*n *= 176)		Odds ratio (95% CI)		*p* value	Adjusted odds ratio (95% CI)		Adjusted *p* value
Preterm delivery—*n* (%)	9	(10.2%)	8	(4.6%)	2.350	(0.874–6.320)	0.083	2.663	(0.820–8.651)	0.103
Post term delivery—*n* (%)	0	(0.0%)	0	(4.6%)	-		-	-		-
Induced labor—*n* (%)	29	(33.3%)	82	(48.0%)	0.543	(0.317–0.929)	0.025	0.571	(0.315–1.036)	0.065
Type of delivery—*n* (%)										
Eutocic	60	(69.0%)	77	(44.5%)	2.771	(1.608–4.775)	< 0.001	3.342	(1.779–6.279)	< 0.001
Instrumental	9	(10.3%)	38	(22.0%)	0.410	(0.188–0.893)	0.022	0.488	(0.203–1.174)	0.109
Cesarean	18	(20.7%)	58	(33.5%)	0.517	(0.282–0.949)	0.032	0.387	(0.195–0.767)	0.007
Intrapartum fever—*n* (%)	1	(1.2%)	22	(12.9%)	0.082	(0.011–0.616)	0.002	0.078	(0.010–0.619)	0.016
Admission to NICU—*n* (%)	8	(9.8%)	17	(9.8%)	0.992	(0.410–2.403)	0.986	0.771	(0.297–2.002)	0.593
Small for gestational age—*n* (%)	11	(12.5%)	12	(7.0%)	1.905	(0.804–4.511)	0.138	2.211	(0.798–6.129)	0.127
Large for gestational age—*n* (%)	2	(2.3%)	11	(6.4%)	0.340	(0.074–1.571)	0.229	0.204	(0.037–1.129)	0.069
Fetal macrosomia—*n* (%)	0	(0.0%)	13	(7.5%)	-		0.005	-		-
Hypertensive diseases of pregnancy—*n* (%)	4	(4.5%)	20	(11.4%)	0.367	(0.122–1.109)	0.066	2.516	(0.724–8.743)	0.147
Gestational hypertension	2	(2.2%)	12	(6.8%)	0.314	(0.069–1.436)	0.116	0.500	(0.096–2.610)	0.411
Preeclampsia	2	(2.2%)	8	(4.5%)	0.483	(0.100–2.323)	0.354	0.415	(0.074–0.342)	0.319
Gestational diabetes—*n* (%)	14	(15.7%)	53	(30.1%)	0.433	(0.225–0.834)	0.011	0.316	(0.151–0.661)	0.002
Premature rupture of membranes—*n* (%)	11	(12.4%)	12	(6.9%)	1.916	(0.809–4.534)	0.134	1.687	(0.618–4.604)	0.308
Fetal growth restriction—*n* (%)	5	(5.6%)	1	(0.6%)	10.357	(1.191–90.057)	0.018	19.213	(1.643–224.694)	0.018
Fetal death—*n* (%)	1	(1.1%)	3	(1.7%)	0.6553	(0.067–6.39)	1.000	0.741	(0.039–13.978)	0.842

^1^Adjustment for the following confounders: BMI before surgery in the bariatric surgery group and BMI before pregnancy in the control group, maternal age, number of previous gestations and abortions, history of active smoking during pregnancy, and essential hypertension. ^2^In addition to previously described confounders, adjustment was also made for gestational age at birth

**Table 4:** Reasons for cesarean delivery

	Bariatric surgery group (*n* = 18)	Control group (*n* = 57)	*p *value
Labor dystocia	1 (5.6%)	17 (29.8%)	0.055
Previous uterine surgery	3 (16.7%)	10 (17.5%)	0.621
Non-reassuring fetal status	4 (22.2%)	9 (15.8%)	0.499
Fetal malpresentation	4 (22.2%)	8 (14.0%)	0.312
Failed induction of labor	3 (16.7%)	9 (15.8%)	1.000
Other causes	3 (16.7%)	4 (7.0%)	-

The frequency of gestational diabetes was lower in the bariatric surgery group (15.7% vs. 30.1%, *p* = 0.002), and, among participants with gestational diabetes, the diagnosis was more likely made in the second trimester in the bariatric surgery group (100.0% vs. 70.2%, *p* = 0.006). No differences were found regarding pharmacological treatment use (Table 5). No statistically significant differences were found regarding hypertensive diseases of pregnancy (bariatric surgery 4.5% vs control 11.4%, *p* = 0.147).

**Table 5:** Time of diagnosis and treatment of gestational diabetes

	Bariatric surgery group (*n* = 14)	Control group (*n* = 53)	*p* value
Time of diagnosis of gestational diabetes—n (%)			
First trimester	0 (0.0%)	20 (37.7%)	0.006
Second trimester	14 (100.0%)	33 (70.2%)	0.006
Gestational diabetes treatment modality			
Lifestyle modifications only	4 (28.6%)	15 (28.3%)	1.000
Metformin	4 (28.6%)	20 (37.7%)	0.755
Insulin therapy	2 (14.3%)	1 (1.9%)	0.108
Metformin and insulin therapy	4 (28.6%)	17 (32.1%)	1.000

Newborn weight, length, and head circumference percentiles were all lower in the bariatric surgery group (*p* < 0.001). There were no significant differences in the prevalence of SGA (12.5% vs. 7.0%, *p* = 0.127) or large for gestational age (LGA) newborns (2.3% vs. 6.4%, *p* = 0.069). Fetal macrosomia was only observed in the control group (0.0% vs. 7.5%, *p* = 0.005), and fetal growth restriction was more frequent in the bariatric surgery group (5.6% vs. 0.6%, *p* = 0.018).

Newborns in the bariatric surgery group had a lower gestational age (39.07 vs. 39.57 weeks, *p* = 0.007). There was no statistically significant difference, however, in the frequency of preterm delivery between (bariatric surgery 10.2% vs. control 4.6%, *p* = 0.103). There were no cases of post-term delivery in either group.

### Bariatric Surgery Group Analysis

Among participants in the bariatric surgery group, no statistically significant differences were observed regarding maternal and fetal outcomes between participants who underwent a gastric bypass or a sleeve gastrectomy (Table 6).

**Fig. 1:**
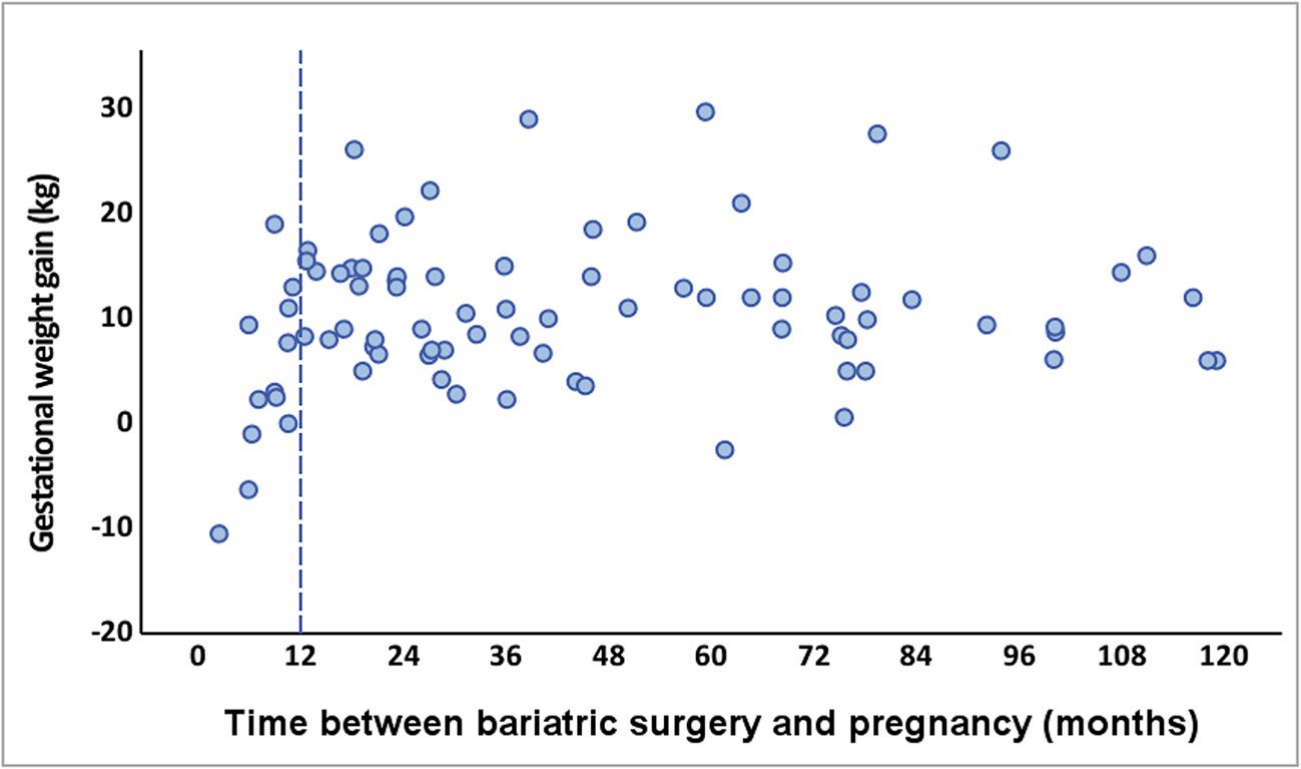
Distribution of gestational weight gain (kg) according to time between surgery and pregnancy (months), in the bariatric surgery group

Participants who became pregnant ≤ 12 months after undergoing bariatric surgery had a lower gestational weight gain (4.18 ± 8.32 vs. 11.73 ± 6.53 kg, *p* = 0.004; Fig. 1). No other statistically significant differences in outcomes were observed regarding time from surgery to pregnancy (Table 6).

**Table 6:** Maternal and fetal outcomes in subgroup analysis

	Type of surgery			Time between surgery and pregnancy			Gestational weight gain		
	Gastric bypass (*n* = 63)	Sleeve gastrectomy (*n* = 26)	*p* value	≤ 12 months (*n* = 12)	> 12 months (*n* = 77)	*p* value	≤ 10 kg (*n* = 43)	> 10 kg (*n* = 41)	*p *value
Gestational weight gain—kg, mean ± SD	10.74 ± 6.98	10.20 ± 7.97	0.758	4.18 ± 8.32	11.73 ± 6.53	0.004	-	-	-
Gestational age at birth—weeks, median (IQR) mean ± SD	39.21 (1.86)	38.64 (2.25)	0.133	39.64 (1.71)	38.92 (1.96)	0.071	38.50 ± 1.72^1^	39.23 ± 1.10^1^	0.022
Preterm delivery—*n* (%)	4 (6.5%)	5 (19.2%)	0.117	0 (0.0%)	9 (11.8%)	0.351	7 (16.7%)	1 (2.5%)	0.031
Induced labor—*n* (%)	10 (38.5%)	19 (31.1%)	0.508	4 (33.3%)	25 (33.3%)	1.000	8 (19.0%)	17 (41.5%)	0.026
Type of delivery—*n* (%)									
Eutocic	42 (68.9%)	18 (69.2%)	0.972	9 (75.0%)	51 (68.0%)	0.747	32 (56.1%)	25 (43.9%)	0.135
Instrumental	8 (13.1%)	1 (3.8%)	0.269	0 (0.0%)	9 (12.0%)	0.350	3 (7.1%)	6 (14.6%)	0.313
Cesarean	11 (18.0%)	7 (26.9%)	0.349	3 (25.0%)	15 (20.0%)	0.707	7 (16.7%)	10 (24.4%)	0.383
Admission to NICU—*n* (%)	4 (7.1%)	4 (15.4%)	0.256	1 (8.3%)	7 (10.0%)	1.000	6 (14.6%)	1 (2.5%)	0.058
Newborn weight—percentile, mean ± SD	35.10 ± 20.69	31.30 ± 20.86	0.562	23.5 (30.5)	34.0 (26.8)	0.222	30.42 ± 21.01	37.68 ± 19.83	0.108
Small for gestational age—*n* (%)	8 (12.9%)	3 (11.5%)	1.000	2 (16.7%)	9 (11.8%)	0.642	1 (2.3%)	1 (2.4%)	1.000
Large for gestational age—*n* (%)	2 (3.2%)	0 (0.0%)	1.000	1 (8.3%)	1 (1.3%)	0.255	7 (16.3%)	3 (7.3%)	0.314
Hypertensive diseases of pregnancy—*n* (%)	2 (3.2%)	2 (7.7%)	0.577	0 (0.0%)	4 (5.2%)	1.000	0 (0.0%)	3 (7.3%)	0.112
Gestational hypertension	1 (1.6%)	1 (3.8%)	0.501	0 (0.0%)	2 (2.6%)	1.000	0 (0.0%)	2 (4.9%)	0.235
Preeclampsia	1 (1.6%)	1 (3.8%)	0.501	0 (0.0%)	2 (2.6%)	1.000	0 (0.0%)	1 (2.4%)	0.488
Gestational diabetes—*n* (%)	11 (17.5%)	3 (11.5%)	0.750	0 (0.0%)	14 (18.2%)	0.201	8 (18.6%)	5 (12.2%)	0.417
Premature rupture of membranes—*n* (%)	7 (11.1%)	4 (15.4%)	0.724	0 (0.0%)	5 (6.5%)	1.000	7 (16.3%)	4 (9.8%)	0.376
Fetal growth restriction—*n* (%)	1 (3.8%)	4 (6.3%)	1.000	1 (8.3%)	10 (13.0%)	1.000	2 (4.7%)	2 (4.9%)	1.000

^1^Mean ± standard deviation. *SD* standard deviation. Statistical significance level was set at *p* < 0.05

Participants in the bariatric surgery group who had a gestational weight gain ≤ 10.0 kg had lower gestational age at birth (38.50 ± 1.72 vs. 39.23 ± 1.10 weeks, *p* = 0.022) and higher risk of preterm delivery (16.7% vs. 2.5%, *p* = 0.031). Additionally, there was a tendency to higher frequency of admission to the neonatal intensive care unit (NICU) (14.6% vs. 2.5%, *p* = 0.058). Participants with a gestational weight gain > 10.0 kg were more likely to have an induced labor (41.5% vs. 19.0%, *p* = 0.026; Table 6).

**Fig. 2:**
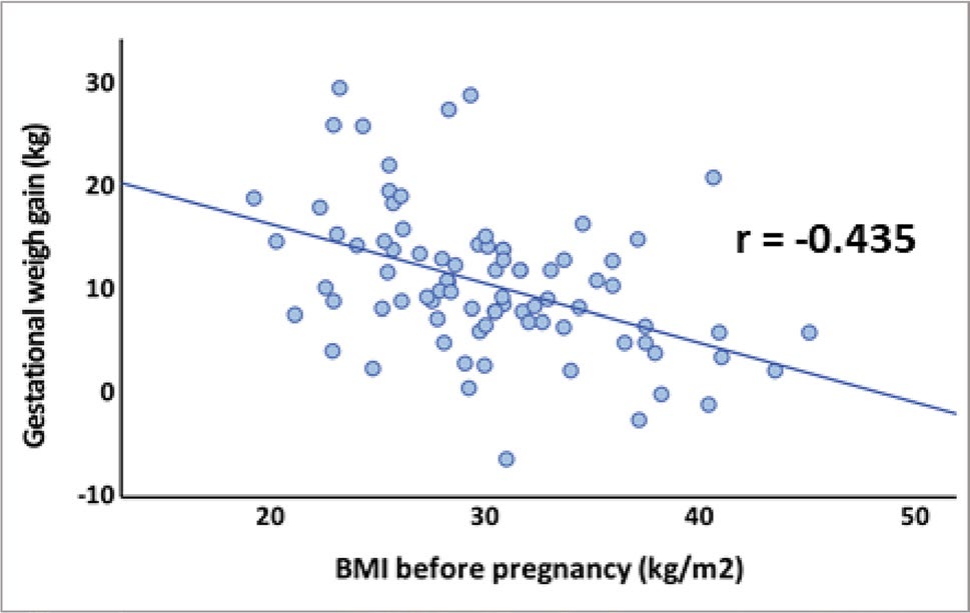
In the bariatric surgery group, gestational weight gain was inversely correlated with BMI before pregnancy

Among participants in the bariatric surgery group, gestational weight gain was inversely correlated with BMI before pregnancy (*r* =  − 0.435, *p* < 0.001; Fig. 2).

## Discussion

### Gestational Diabetes

In our study, pregnancy after bariatric surgery was associated with a lower incidence of gestational diabetes. These findings are consistent with previous studies [10, 24–27] and could be attributable to two main changes that occur after bariatric surgery: the first being weight loss, as obesity is a well-known risk factor for the development of gestational diabetes, and the second the changes that occur in glucose metabolism after bariatric surgery, independently of weight loss [11]. Previous studies have shown that bariatric surgery leads to improved fasting glucose and exaggerated postprandial insulin response, which in some cases leads to postprandial hypoglycemia, and that these changes in glucose metabolism persist during pregnancy [28–30].

Additionally, among patients with gestational diabetes, previous bariatric surgery was associated with a diagnosis later in pregnancy, implying that these patients were exposed to hyperglycemia for a shorter period. To the authors’ knowledge, there is limited existing evidence regarding the effect of bariatric surgery on the timing of diagnosis of gestational diabetes, and the results in our study could have been influenced by the different diagnostic methods used in the second trimester in the two groups.

### Newborn Anthropometry

In our study, newborns of postbariatric pregnancies were smaller; there was a higher risk of fetal growth restriction and lower risk of fetal macrosomia. Likewise, previous studies have shown that pregnancy after bariatric surgery is associated with a lower risk of LGA newborns, and higher risk of SGA newborns and fetal growth restriction [10, 12, 24, 27, 31]. Similarly, weight loss and glucose metabolism changes produced by bariatric surgery seem to play a major role in determining newborn size [11, 28, 32]. Other proposed mechanisms include maternal deficiency in micronutrients and protein and changes in inflammatory cytokines and cellular oxidative stress [11, 33]. Low cord blood IGF1 and leptin levels in infants of mothers with a history of gastric bypass have also been reported [34].

### Delivery Type

Although there is conflicting evidence regarding the effect of bariatric surgery on delivery type, it appears that women who become pregnant after bariatric surgery have a higher risk of cesarean delivery compared to healthy controls [35–37] and lower risk compared to controls with obesity [38, 39], as was the case for our study. Higher maternal age and higher prevalence of previous cesarean delivery could explain the higher risk of cesarean delivery reported in some studies [9, 40, 41]. Caregiver bias has also been proposed as a contributing factor [35, 42]. On the other hand, lower BMI and lower incidence of gestational diabetes and LGA neonates after bariatric surgery could contribute to a lower risk of cesarean delivery[40, 43]. A lower risk of labor dystocia in postbariatric pregnancies, possibly in relation to smaller neonates, has also been reported [9, 41].

### Gestational Weight Gain

Evidence regarding the effect of bariatric surgery on gestational weight gain is conflicting. While most studies suggest that BG leads to lower gestational weight gain [9, 44–46], others point to similar or even higher gestational weight gain [10, 47]. These disparities could be due, in part, to differences in the characteristics of control groups in each study.

In women without history of bariatric surgery, excessive gestational weight gain is known to increase the risk of LGA neonates, cesarean delivery, gestational diabetes, and hypertensive diseases of pregnancy [48]. In our study, pregnancy after bariatric surgery was associated with higher gestational weight gain, compared to controls with BMI ≥ 35 kg/m^2^, but there was no increase in the above-mentioned outcomes. In addition, insufficient gestational weight gain is known to increase the risk of preterm birth and SGA neonates [48], and our study confirmed that in postbariatric pregnancies gestational weight gain < 10.0kg leads to higher risk of preterm birth. Similarly, in a bicentric retrospective study conducted by Grandfils et al., postbariatric pregnancies with inadequate gestational weight gain were at higher risk of preterm delivery [49]. Furthermore, Stentebjerg et al. found that women who conceive within 18 months after surgery have less gestational weight gain, which is consistent our results regarding timing of pregnancy and gestational weight gain.

In the authors’ opinion, some explanations for the higher gestational weight gain observed in the bariatric surgery group overall could be that the dietary plan prescribed to pregnant women in our center is of similar nutritional value across different prepregancy BMIs, and the fact that women with a higher prepregancy BMI are actively encouraged to avoid excessive gestational weight gain [50].

Importantly, there is still debate regarding the ideal gestational weight gain in postbariatric pregnancies, and whether current guidelines can be applied to this specific population[14, 49].

Due to conflicting evidence in the literature, in our center, women who undergo bariatric surgery are encouraged to wait a minimum of 12 months before becoming pregnant, and subsequently to maintain an adequate gestational weight gain, in accordance to the 2009 Institute of Medicine guidelines [50].

### Study Limitations

The retrospective and observational nature of the study limits its interpretation. Also, the sample size might have been insufficient to detect differences between groups in some outcomes. This could explain some disparities in our results and the literature.

In our study, adjustment was made for BMI before surgery in the bariatric surgery group, and BMI before pregnancy in the control group. Only women with a BMI of ≥ 35 kg/m^2^ were included in the control group, as they would be considered bariatric surgery candidates. This was done, as in other studies, to assess the effect of this procedure on women with a BMI ≥ 35 kg/m^2^, who presumably would have carried out future pregnancies with a similar BMI if not for the surgery [10, 41, 47]. This assumption could, however, limit the interpretation of the results, as some pregnancies occur several years after bariatric surgery, and weight changes could happen during this time interval.

Finally, another limitation is the fact that micronutrient status during pregnancy was not evaluated. Bariatric surgery is known to increase the risk of micronutrient deficiencies [34, 51], and nutritional status of the mother seems to play a role in fetal development [33]. There is still limited evidence, however, on the impact of micronutrient deficiencies in postbariatric pregnancies on perinatal outcomes.

## Conclusion

Pregnancy after bariatric surgery is associated with both risks and benefits, which should be taken into consideration and discussed with women of fertile age considering undergoing such procedure. In case of pregnancy after bariatric surgery, individualized care, ideally in a specialized center, is necessary to ensure adequate gestational weight gain and avoid micronutrient deficiencies.

Further research should focus on the ideal timing between surgery and pregnancy and ideal gestational weight gain in these patients.

## Data Availability

The dataset for this study is available from the corresponding author on reasonable request and ethical approval.

## References

[1] Leddy MA, Power ML, Schulkin J (2008). The impact of maternal obesity on maternal and fetal health. Rev Obstet Gynecol.

[2] Poston L, Caleyachetty R, Cnattingius S, Corvalán C, Uauy R, Herring S (2016). Preconceptional and maternal obesity: epidemiology and health consequences. Lancet Diabetes Endocrinol.

[3] Drake AJ, Reynolds RM (2010). Focus on obesity: Impact of maternal obesity on offspring obesity and cardiometabolic disease risk. Reproduction.

[4] Shankar K, Harrell A, Liu X, Gilchrist JM, Ronis MJ, Badger TM (2008). Maternal obesity at conception programs obesity in the offspring. Am J Physiol Regul Integr Comp Physiol.

[5] Jakobsen GS, Småstuen MC, Sandbu R, Nordstrand N, Hofsø D, Lindberg M (2018). Association of bariatric surgery vs medical obesity treatment with long-term medical complications and obesity-related comorbidities. JAMA.

[6] Arterburn DE, Telem DA, Kushner RF, Courcoulas AP (2020). Benefits and risks of bariatric surgery in adults: a review. JAMA.

[7] Wendy A Brown SS, Ronald Liem, Jennifer Holland. Seventh IFSO Global Registry Report 2022. 2022.

[8] Obstetricians ACo, Gynecologists (2009). Bariatric surgery and pregnancy. ACOG practice bulletin no. 105. Obstet Gynecol.

[9] Fisher SA, Stetson BT, Kominiarek MA (2023). Pregnancy After Bariatric Surgery. JAMA.

[10] Johansson K, Cnattingius S, Näslund I, Roos N, Trolle Lagerros Y, Granath F (2015). Outcomes of pregnancy after bariatric surgery. N Engl J Med.

[11] Falcone V, Stopp T, Feichtinger M, Kiss H, Eppel W, Husslein PW (2018). Pregnancy after bariatric surgery: a narrative literature review and discussion of impact on pregnancy management and outcome. BMC Pregnancy Childbirth.

[12] Akhter Z, Rankin J, Ceulemans D, Ngongalah L, Ackroyd R, Devlieger R (2019). Pregnancy after bariatric surgery and adverse perinatal outcomes: a systematic review and meta-analysis. PLoS Med.

[13] Rottenstreich A, Elazary R, Goldenshluger A, Pikarsky AJ, Elchalal U, Ben-Porat T (2019). Maternal nutritional status and related pregnancy outcomes following bariatric surgery: a systematic review. Surg Obes Relat Dis.

[14] Shawe J, Ceulemans D, Akhter Z, Neff K, Hart K, Heslehurst N (2019). Pregnancy after bariatric surgery: consensus recommendations for periconception, antenatal and postnatal care. Obes Rev.

[15] Beard JH, Bell RL, Duffy AJ (2008). Reproductive considerations and pregnancy after bariatric surgery: current evidence and recommendations. Obes Surg.

[16] Alamri SH, Abdeen GN (2022). Maternal nutritional status and pregnancy outcomes post-bariatric surgery. Obes Surg.

[17] Bilardo CM, Chaoui R, Hyett JA, Kagan KO, Karim JN, Papageorghiou AT (2023). ISUOG Practice Guidelines (updated): performance of 11–14-week ultrasound scan. Ultrasound Obstet Gynecol.

[18] Organization WH. Diagnostic criteria and classification of hyperglycaemia first detected in pregnancy. World Health Organization 2013 24199271

[19] ACOG Practice Bulletin No (2019). 203: Chronic hypertension in pregnancy. Obstet Gynecol.

[20] ACOG Practice Bulletin No (2019). 202 Summary: gestational hypertension and preeclampsia. Obstet Gynecol.

[21] Lees CC, Stampalija T, Baschat A, da Silva CF, Ferrazzi E, Figueras F (2020). ISUOG Practice Guidelines: diagnosis and management of small-for-gestational-age fetus and fetal growth restriction. Ultrasound Obstet Gynecol.

[22] Salomon LJ, Alfirevic Z, Da Silva CF, Deter RL, Figueras F, Ghi T (2019). ISUOG practice guidelines: ultrasound assessment of fetal biometry and growth. Ultrasound Obstet Gynecol.

[23] Myers ER, Sanders GD, Coeytaux RR, et al. AHRQ Comparative Effectiveness Reviews. Labor Dystocia. Rockville (MD): Agency for Healthcare Research and Quality (US); 2020

[24] Galazis N, Docheva N, Simillis C, Nicolaides KH (2014). Maternal and neonatal outcomes in women undergoing bariatric surgery: a systematic review and meta-analysis. Eur J Obstet Gynecol Reprod Biol.

[25] Maggard MA, Yermilov I, Li Z, Maglione M, Newberry S, Suttorp M (2008). Pregnancy and fertility following bariatric surgery: a systematic review. JAMA.

[26] Vrebosch L, Bel S, Vansant G, Guelinckx I, Devlieger R (2012). Maternal and neonatal outcome after laparoscopic adjustable gastric banding: a systematic review. Obes Surg.

[27] Yi XY, Li QF, Zhang J, Wang ZH (2015). A meta-analysis of maternal and fetal outcomes of pregnancy after bariatric surgery. Int J Gynecol Obstet.

[28] Feichtinger M, Stopp T, Hofmann S, Springer S, Pils S, Kautzky-Willer A (2017). Altered glucose profiles and risk for hypoglycaemia during oral glucose tolerance testing in pregnancies after gastric bypass surgery. Diabetologia.

[29] Goldfine AB, Mun E, Devine E, Bernier R, Baz-Hecht M, Jones D (2007). Patients with neuroglycopenia after gastric bypass surgery have exaggerated incretin and insulin secretory responses to a mixed meal. J Clin Endocrinol Metab.

[30] Göbl CS, Bozkurt L, Tura A, Leutner M, Andrei L, Fahr L (2017). Assessment of glucose regulation in pregnancy after gastric bypass surgery. Diabetologia.

[31] Chevrot A, Kayem G, Coupaye M, Lesage N, Msika S, Mandelbrot L (2016). Impact of bariatric surgery on fetal growth restriction: experience of a perinatal and bariatric surgery center. Am J Obstet Gynecol.

[32] Scholl TO, Sowers M, Chen X, Lenders C (2001). Maternal glucose concentration influences fetal growth, gestation, and pregnancy complications. Am J Epidemiol.

[33] De Bernabé JV, Soriano T, Albaladejo R, Juarranz M, Mae Calle, Martínez D,  (2004). Risk factors for low birth weight: a review. Eur J Obstet Gynecol Reprod Biol.

[34] Gascoin G, Gerard M, Sallé A, Becouarn G, Rouleau S, Sentilhes L (2017). Risk of low birth weight and micronutrient deficiencies in neonates from mothers after gastric bypass: a case control study. Surg Obes Relat Dis.

[35] Sheiner E, Levy A, Silverberg D, Menes TS, Levy I, Katz M (2004). Pregnancy after bariatric surgery is not associated with adverse perinatal outcome. Am J Obstet Gynecol.

[36] Belogolovkin V, Salihu HM, Weldeselasse H, Biroscak BJ, August EM, Mbah AK (2012). Impact of prior bariatric surgery on maternal and fetal outcomes among obese and non-obese mothers. Arch Gynecol Obstet.

[37] Lapolla A, Marangon M, Dalfrà MG, Segato G, De Luca M, Fedele D (2010). Pregnancy outcome in morbidly obese women before and after laparoscopic gastric banding. Obes Surg.

[38] Stephansson O, Johansson K, Söderling J, Näslund I, Neovius M (2018). Delivery outcomes in term births after bariatric surgery: population-based matched cohort study. PLoS Med.

[39] Burke AE, Bennett WL, Jamshidi RM, Gilson MM, Clark JM, Segal JB (2010). Reduced incidence of gestational diabetes with bariatric surgery. J Am Coll Surg.

[40] Witt WP, Wisk LE, Cheng ER, Mandell K, Chatterjee D, Wakeel F (2015). Determinants of cesarean delivery in the US: a lifecourse approach. Matern Child Health J.

[41] Weintraub AY, Levy A, Levi I, Mazor M, Wiznitzer A, Sheiner E (2008). Effect of bariatric surgery on pregnancy outcome. Int J Gynecol Obstet.

[42] Kominiarek MA, editor Preparing for and managing a pregnancy after bariatric surgery. Seminars in perinatology 2011 Elsevier 10.1053/j.semperi.2011.05.022 PMC3345131 22108087

[43] Ehrenberg HM, Durnwald CP, Catalano P, Mercer BM (2004). The influence of obesity and diabetes on the risk of cesarean delivery. Am J Obstet Gynecol.

[44] Gagnon G, Carreau A-M, Cloutier-Langevin C, Plante A-S, Weisnagel SJ, Marceau S (2022). Trimester-specific gestational weight gain in women with and without previous bariatric surgeries. Eur J Obstet Gynecol Reprod Biol.

[45] Berglind D, Willmer M, Näslund E, Tynelius P, Sørensen T, Rasmussen F (2014). Differences in gestational weight gain between pregnancies before and after maternal bariatric surgery correlate with differences in birth weight but not with scores on the body mass index in early childhood. Pediatr Obes.

[46] Santulli P, Mandelbrot L, Facchiano E, Dussaux C, Ceccaldi P-F, Ledoux S (2010). Obstetrical and neonatal outcomes of pregnancies following gastric bypass surgery: a retrospective cohort study in a French referral centre. Obes Surg.

[47] Iacovou C, Maric T, Bourke M, Patel D, Savvidou M (2023). Gestational weight gain in pregnancies following bariatric surgery. Obes Surg.

[48] Goldstein RF, Abell SK, Ranasinha S, Misso M, Boyle JA, Black MH (2017). Association of gestational weight gain with maternal and infant outcomes: a systematic review and meta-analysis. JAMA.

[49] Grandfils S, Demondion D, Kyheng M, Duhamel A, Lorio E, Pattou F (2019). Impact of gestational weight gain on perinatal outcomes after a bariatric surgery. J Gynecol Obstet Hum Reprod.

[50] Pregnancy WGD (2009). Reexamining the guidelines.

[51] Shankar P, Boylan M, Sriram K (2010). Micronutrient deficiencies after bariatric surgery. Nutrition.

